# Approved Societies and Hospital Treatment

**Published:** 1922-06

**Authors:** P. J. Michelli

**Affiliations:** Secretary, Seamen's Hospital, Greenwich, S.E.10.


					To the Editor of The Hospital and Health Review.
Sir,?The last issue of The Hospital and Health
Review contained an excellent article by Mr. F.
Squires, which is an admirable guide for the hospital
secretary. As soon as it was published I notified the
Hearts of Oak; of a man lying in this hospital, and
have received a reply stating that that Approved
Society has disposed of its assets in the form of
additional cash benefits to its members, and cannot
pay anything for the patient lying in our wards. I
therefore think that others may like to know, so that
they may be saved the trouble of writing to the
National Amalgamated Society in respect of patients
insured in the Hearts of Oak.
I am, Sir,
Yours, &c.,
P. J. Michelu,
Secretary, Seamen's Hospital, Greenwich, S.E.10.

				

